# Population pharmacokinetics of mefloquine, piperaquine and artemether-lumefantrine in Cambodian and Tanzanian malaria patients

**DOI:** 10.1186/1475-2875-12-235

**Published:** 2013-07-10

**Authors:** Eva Maria Staehli Hodel, Monia Guidi, Boris Zanolari, Thomas Mercier, Socheat Duong, Abdunoor M Kabanywanyi, Frédéric Ariey, Thierry Buclin, Hans-Peter Beck, Laurent A Decosterd, Piero Olliaro, Blaise Genton, Chantal Csajka

**Affiliations:** 1 Swiss Tropical and Public Health Institute, University of Basel, Basel, Switzerland; 2 School of Pharmaceutical Sciences, University of Geneva, University of Lausanne, Geneva, Switzerland; 3 Division of Clinical Pharmacology and Toxicology, Department of Laboratories, Centre Hospitalier Universitaire Vaudois and University of Lausanne, Lausanne, Switzerland; 4 National Center for Parasitology, Entomology and Malaria Control, Phnom Penh, Cambodia; 5 Ifakara Health Institute, Dar es Salaam, Tanzania; 6 Institut Pasteur in Cambodia, Molecular Epidemiology UnitInstitut Pasteur in Cambodia, Molecular Epidemiology Unit, Phnom Penh, Cambodia; 7 UNICEF/UNDP/World Bank/WHO Special Programme for Research and Training in Tropical Diseases (TDR), Geneva, Switzerland; 8 Department of Ambulatory Care and Community Medicine & Division of Infectious Diseases, University Hospital, Lausanne, Switzerland

**Keywords:** Malaria, Population pharmacokinetics, Artemisinin-based combination therapy, Tanzania, Cambodia, Piperaquine, Mefloquine, Lumefantrine, Artemether, Nonlinear mixed-effects modelling

## Abstract

**Background:**

Inter-individual variability in plasma concentration-time profiles might contribute to differences in anti-malarial treatment response. This study investigated the pharmacokinetics of three different forms of artemisinin combination therapy (ACT) in Tanzania and Cambodia to quantify and identify potential sources of variability.

**Methods:**

Drug concentrations were measured in 143 patients in Tanzania (artemether, dihydroartemisinin, lumefantrine and desbutyl-lumefantrine), and in 63 (artesunate, dihydroartemisinin and mefloquine) and 60 (dihydroartemisinin and piperaquine) patients in Cambodia. Inter- and intra-individual variabilities in the pharmacokinetic parameters were assessed and the contribution of demographic and other covariates was quantified using a nonlinear mixed-effects modelling approach (NONMEM^®^).

**Results:**

A one-compartment model with first-order absorption from the gastrointestinal tract fitted the data for all drugs except piperaquine (two-compartment). Inter-individual variability in concentration exposure was about 40% and 12% for mefloquine. From all the covariates tested, only body weight (for all antimalarials) and concomitant treatment (for artemether only) showed a significant influence on these drugs’ pharmacokinetic profiles. Artesunate and dihydroartemisinin could not be studied in the Cambodian patients due to insufficient data-points. Modeled lumefantrine kinetics showed that the target day 7 concentrations may not be achieved in a substantial proportion of patients.

**Conclusion:**

The marked variability in the disposition of different forms of ACT remained largely unexplained by the available covariates. Dosing on body weight appears justified. The concomitance of unregulated drug use (residual levels found on admission) and sub-optimal exposure (variability) could generate low plasma levels that contribute to selecting for drug-resistant parasites.

## Background

Artemisinin-based combination therapy (ACT) is the current first-line treatment of malaria [1]. At present, the forms of ACT recommended by the World Health Organization (WHO) contain artemether (AM) plus lumefantrine (LF), artesunate (AS) plus either amodiaquine (AQ), mefloquine (MQ), pyronaridine (PN) or sulphadoxine-pyrimethamine (SP), and dihydroartemisinin (DHA) plus piperaquine (PPQ) [1]. While hundreds of thousand courses of ACT are deployed each year [2], there is a limited number of studies measuring levels of drug exposure and relating it to treatment efficacy and safety.

Acquiring this information is paramount in order to optimize treatment and, especially, prevent resistance which may result from inadequate dosing. One of the main questions is to know if giving the recommended dose produces the same level of exposure in all, or otherwise what proportion or categories of subjects, and under which circumstances, would be systematically over- or under-dosed.

In other words, one needs to know if dosing regimens are adequate or if there are systematic dosing errors in which populations, especially on account of inter-subject variability and special groups like children and pregnant women. There is evidence that SP was systematically under-dosed in children and that the lower drug levels have contributed to the emergence of parasite resistance to this drug [3,4]. The situation is further complicated by the fact that the target doses and therapeutic windows have been established based mostly on data in adults, and assume all patients require the same level of exposure, while, for instance, the contribution of immunity to parasite clearance will change with age and malaria transmission.

In this respect, one will need to know how the pharmacokinetics contributes to efficacy or safety outcomes. Examples of proposed surrogate efficacy correlates are day 7 drug plasma concentrations for LF [5-7] and the time for drug plasma concentrations to fall below 500 μg/l (the minimal inhibitory concentration, MIC) for MQ [8].

Treating with a wrong dose may have both individual and general consequences. Over-exposure increases the risk of toxicity; under-dosing may lead to treatment failure, but also carries the risk of selecting for drug-resistant parasites, which can spread to the rest of the population [9-12].

Two clinical trials found that AM-LF was highly efficacious in Tanzania, but much less effective (71% cure rate) in Cambodia [13,14]. The cure rate in Cambodia increased to 86.5% in the subsequent years when 250 ml milk and coconut biscuits were provided with each dose of the study medication to increase drug absorption [13]. These findings raised the question of the factors that could have contributed to the lower efficacy of AM-LF in the Cambodian population. Parasite susceptibility is indeed a potential explanation [15-24], although no known molecular marker exists at the moment. Another possibility is that differences in drug levels induced by genetic or other factors could explain the difference in drug response between these two populations.

The objectives of this paper were to characterize the population pharmacokinetics of AS, DHA, MQ and PPQ in Cambodian patients and AM and LF in Tanzanian patients and to identify demographic and other factors that could explain variability in drug levels. In addition, day 7 concentrations have been shown to be a good surrogate marker of treatment success and model based-simulations of LF were performed to predict the proportion of patients with concentrations below the proposed day 7 cut-off values.

## Methods

### Study area, patients and data

**Figure 1:**
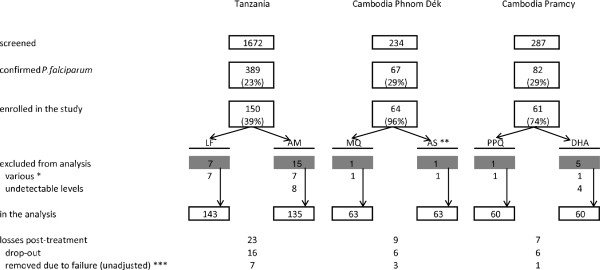
**Study profiles.** * Tanzania: hemoglobin <5.0 g/dL (2 patients), unable to swallow drug (1 patient), withdrawal of consent (2 patients), blood withdrawal not possible (1 patient), >9’999 parasites per 200 white blood cells (1 patient). Cambodia: withdrawal of consent (1 patient in each study). ** In 4 patients only DHA but no AS could be detected. *** Tanzania: 4 late clinical failures, 3 late parasitological failures and 1 late clinical and parasitological failure. Cambodia: 3 late parasitological failures in Phnom Dék and 1 late clinical failure in Pramoy. AM: artemether; AS: artesunate; DHA: dihydroartemisinin; LF: lumefantrine; MQ: mefloquine; PPQ: piperaquine.

Three studies have been conducted, one in Tanzania and two in Cambodia. The study profiles are described in Figure 1. The first study was performed during March to May 2008 at the Kibaoni Health Centre, Kilombero district, Morogoro region, Tanzania. A total of 1,672 patients with suspected malaria were screened by rapid diagnostic test (Paracheck, Orchid Biomedical Systems, India) and 389 (23%) had parasitologically-proven *Plasmodium falciparum* malaria (confirmed and quantified by microscopy). After giving their informed consent to participate, patients were included in the study if they did not present signs of complicated malaria or any other severe concomitant illness. Six AM-LF (Coartem^®^, Novartis Pharma, Switzerland) doses were administered at time 0, 8, 20, 32, 44 and 56 h according to body weight (see Table 1); patients were either admitted for three days or asked to come back to the health facility for each drug administration. Mothers of breastfed patients were encouraged to feed their children and patients admitted were provided with food. Patients who reported that they have not eaten within two hours to prior dose intake were instructed to eat as soon as possible. Patients were seen by the clinical officer on days 0, 1, 2, 3, 7, 14, 28, and 42. A blood sample for pharmacokinetic measurement was taken at baseline (pre-dose on day 0) and on days 1, 2, and 7 at pre-defined random times after the last dosing. At each visit, a filter paper sample and a thin and thick smear were taken, in addition to axillary temperature, respiratory rate and haemoglobin measurements (only on days 0, 28 and 42) as well as evaluation of symptoms (e.g. headache, vomiting, and diarrhea). The exact dose and time of last drug intake, body weight, height, age, sex, food intake and concomitant medications were carefully recorded. If patients suffered from concomitant illnesses they were provided with additional treatment (paracetamol, mebendazole, metronidazole, cloxacillin, amoxicillin).

**Table 1:** Dosing regimens of the study drugs

**Drug**	**Body weight [kg]**	**Age [years]**	**Day 0**	**Day 1**	**Day 2**
AM-LF	5–14		AM: 2 × 20 mg	AM: 2 × 20 mg	AM: 2 × 20 mg
			LF: 2 × 120 mg	LF: 2 × 120 mg	LF: 2 × 120 mg
	15–24		AM: 2 × 40 mg	AM: 2 × 40 mg	AM: 2 × 40 mg
			LF: 2 × 240 mg	LF: 2 × 240 mg	LF: 2 × 240 mg
	25–34		AM: 2 × 60 mg	AM: 2 × 60 mg	AM: 2 × 60 mg
			LF: 2 × 360 mg	LF: 2 × 360 mg	LF: 2 × 360 mg
	≥35		AM: 2 × 80 mg	AM: 2 × 80 mg	AM: 2 × 80 mg
			LF: 2 × 480 mg	LF: 2 × 480 mg	LF: 2 × 480 mg
AS-MQ	10–12.5		AS: 50 mg	AS: 50 mg	AS: 50 mg
			MQ: 125 mg	MQ: 125 mg	
	13–15.5		AS: 50 mg	AS: 50 mg	AS: 50 mg
			MQ: 125 mg	MQ: 125 mg	MQ: 125 mg
	16–24.5		AS: 100 mg	AS: 100 mg	AS: 100 mg
			MQ: 250 mg	MQ: 250 mg	
	25–34.5		AS: 150 mg	AS: 150 mg	AS: 150 mg
			MQ: 250 mg	MQ: 250 mg	MQ: 250 mg
	35–37		AS: 200 mg	AS: 200 mg	AS: 200 mg
			MQ: 250 mg	MQ: 250 mg	MQ: 250 mg
	38–57		AS: 200 mg	AS: 200 mg	AS: 200 mg
			MQ: 500 mg	MQ: 500 mg	MQ: 250 mg
	58–76		AS: 200 mg	AS: 200 mg	AS: 200 mg
			MQ: 500 mg	MQ: 500 mg	MQ: 500 mg
DHA-PPQ		6–11	DHA: 60 mg	DHA: 60 mg	DHA: 40 mg
			PPQ: 480 mg	PPQ: 480 mg	PPQ: 320 mg
		11–16	DHA: 80 mg	DHA: 80 mg	DHA: 80 mg
			PPQ: 640 mg	PPQ: 640 mg	PPQ: 640 mg
		>16	DHA: 120 mg	DHA: 120 mg	DHA: 80 mg
			PPQ: 960 mg	PPQ: 960 mg	PPQ: 640 mg

*Abbreviations: AM* artemether, *AS* artesunate, *DHA* dihydroartemisinin, *LF* lumefantrine, *MQ* mefloquine, *PPQ* piperaquine.

The second study was conducted during October 2007 to February 2008 at the Phnom Dék Health Centre, Rovieng district, Preah Vihear province, Cambodia. Entry criteria and study procedures were identical as in the Tanzanian study with minor adaptations described below. In total, 234 suspected malaria cases were screened by microscopy, of whom 67 (29%) were found to be infected with *P. falciparum* and 74 (32%) with *Plasmodium vivax* (no mixed infections were detected). Pregnant or lactating women were excluded. Three doses of AS (Arsumax^®^, Sanofi-Aventis, France) and MQ (Eloquine^®^, Medochemie Ltd, Cyprus) were given according to body weight on three consecutive days (see Table 1). Patients were seen by the clinical officer on days 1, 2, 3, 7, 14, 21, 28, 35 and 42. Sampling for the pharmacokinetic study was done at pre-dose and approximately 1 h after first dose intake on day 0, and on days 1, 2, 7 and 14 at pre-defined random times after drug intake.

The third study was performed during July to October 2008 at Pramoy Health Centre, Veal Veng district, Pursat province, Cambodia. A total of 287 suspected malaria cases were screened by microscopy, of whom 82 (29%) were infected with *P. falciparum* and 50 (17%) with *P. vivax* (no mixed infections). Children younger than six years of age and pregnant or lactating women were excluded. Three doses of DHA-PPQ (Duo-Cotecxin^®^, Zhejiang Holley Nanhu Pharmaceutical Co., Ltd, China) were given according to age (as per national guidelines) on three consecutive days (see Table 1). Same follow-up and blood sampling as at Phnom Dék were performed.

### Laboratory methods

Samples of 2 ml of venous blood were collected on an EDTA Vacutainer and kept on ice for no longer than 6 h after withdrawal and then aliquoted into whole blood, plasma and pellet and immediately stored in liquid nitrogen or a –80°C freezer. Plasma concentrations of 14 antimalarial drugs and their metabolites, i.e. AM, AS, DHA, amodiaquine, *N*-desethyl-amodiaquine, LF, desbutyl-lumefantrine (DLF), PPQ, PN, MQ, chloroquine, quinine and SP, were determined simultaneously using a liquid chromatography–tandem mass spectrometry method (LC–MS/MS) previously reported [25]. The method was validated according to FDA recommendations, including assessment of extraction yield, matrix effect variability, overall process efficiency, standard addition experiments as well as anti-malarials short- and long-term stability in plasma. The method is precise (inter-day coefficient of variation: 3.1–12.6%) and sensitive (lower limits of quantification 0.15–3.0 for basic/neutral anti-malarials and 0.75–5 ng/ml for artemisinin derivatives, respectively). The laboratory is part of the quality control system of the World-Wide Antimalarial Resistance Network (WWARN).

Pharmacogenetic profiles of the patients were generated using a sequencing strategy [26]. Detailed results of the population genetic analysis are presented elsewhere [27].

### Model-based pharmacokinetic analysis

The pharmacokinetic analysis for each drug taken separately was performed using the NONMEM computer program [28] Version 6 (NM-TRAN version II). It uses mixed (fixed and random) effects regression to estimate population means and variances of the pharmacokinetic parameters and to identify factors that influence them.

#### Structural model

**Figure 2:**
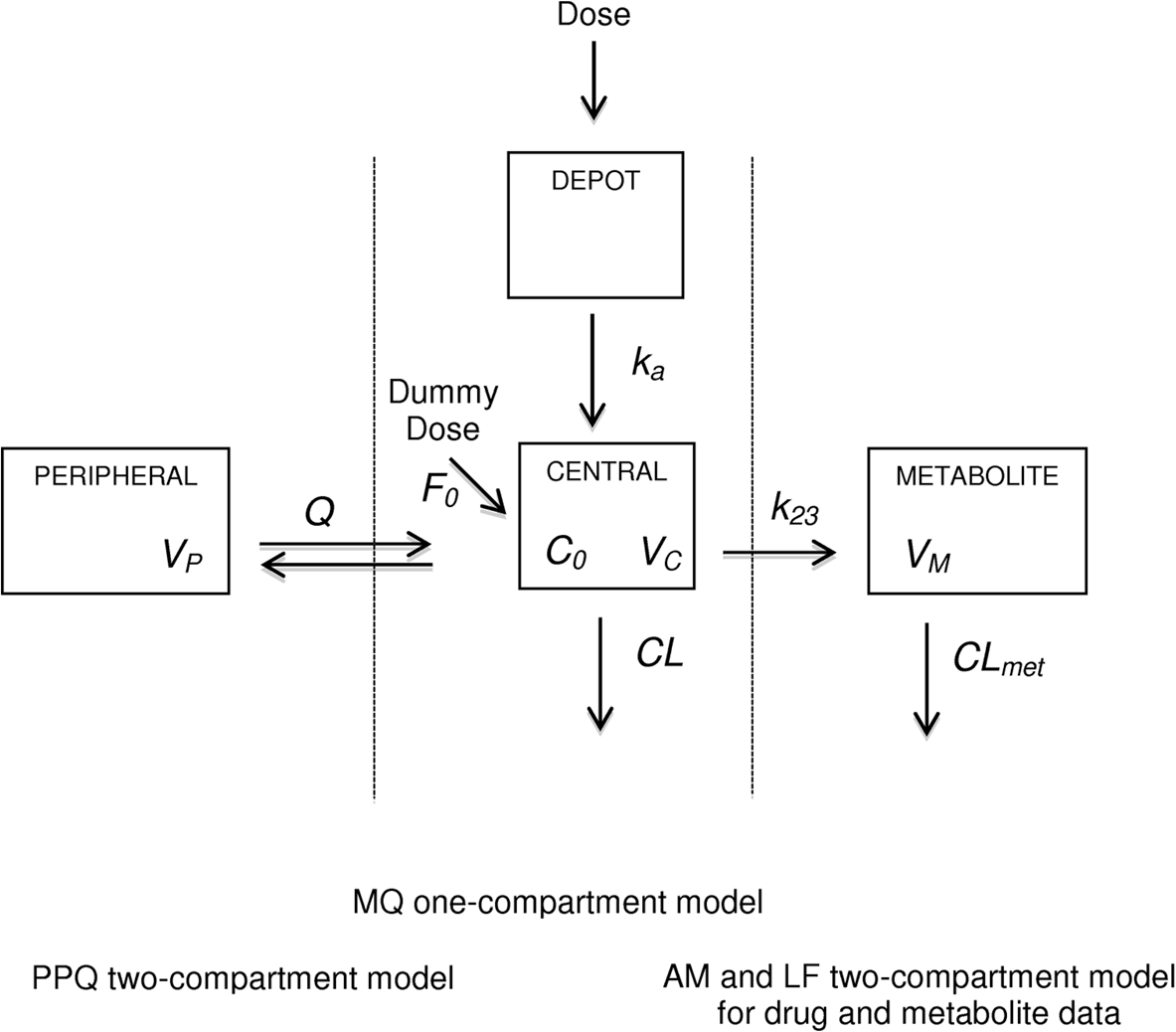
**Models used to describe AM, LF, MQ and PPQ and active metabolites DHA and DLF.** For AM and LF, *CL* = (*k*_*20*_*– k*_*23*_) × *V*_*C*_, with *k*_*20*_ = *CL/V*_*C*_. Because of problems of identification of *k*_*23*_, *V*_*M*_ and *CL*_*met*_, *V*_*M*_ was assumed to equal *V*_*C*_. The concentration at baseline (*C*_*0*_) was fitted by using a dummy dose of 1 mg times the estimated parameter *F*_*0*_ (see text). AM: artemether; AS: artesunate; DHA: dihydroartemisinin; DLF: desbutyl-lumefantrine; LF: lumefantrine; MQ: mefloquine; PPQ: piperaquine.

One-, two- and three-compartment pharmacokinetic models with first-order absorption, with and without absorption lag times, were compared. Additional one or two-compartments were used for anti-malarials presenting metabolite concentrations (AM and LF). The final parameters estimated were systemic clearance (*CL/F*), inter-compartmental clearance (*Q/F*), central volume of distribution (*V*_*C*_*/F*), peripheral volume of distribution (*V*_*P*_*/F*) and absorption rate constant (*k*_a_). Since no intravenous drug concentration data were available, these parameters represent apparent values. Where available, metabolite data were included into the model and metabolism rate constant from drug compartment to metabolite compartment (*k*_*23*_) and metabolite clearance (*CL*_*met*_) were also estimated. Owing to identifiability problems, the volume of distribution of the metabolites (*V*_*M*_) DLF and DHA were assumed to equal LF and AM *V*_*C*_, respectively. Analysis of baseline plasma samples (i.e. day 0 prior treatment) showed that some patients had non-zero concentration of the drug, probably resulting from the treatment of the previous malaria episode or intake of non-declared drugs [29,30]. The observed baseline residual plasma concentrations were fitted by estimating a factor (*F*_0_) that provided an estimation of the residual doses from previous treatment. A schematic representation of the models is presented in Figure 2.

#### Statistical model

Exponential errors following a log-normal distribution were assumed for the description of inter-patient variability of the pharmacokinetic parameters and were of the form: $\theta_{i}=\theta \times e^{\eta_{j}}$ where *θ*_*i*_ is the individual pharmacokinetic parameter value in the *j*^th^ individual, *θ* is the population parameter estimate, and *η*_*j*_ is the random effect value, which is independently and normally distributed with a mean of 0 and variance *ω*^*2*^. Proportional and combined proportional-and-additive error models were compared to describe intra-patient (residual) variability for the mother compound, and if available for its metabolite using *C*_*pij*_(1 + *ϵ*_1*ij*_) + *ϵ*_2*ij*_ or *C*_*mij*_(1 + *ϵ*_3*ij*_) + *ϵ*_4*ij*_, where *C*_*pij*_ are the corresponding predicted *i*^th^ drug plasma concentration and *C*_*mij*_ are the predicted metabolite concentration for the *j*^th^ individual, and *ϵ*_1*ij*_, *ϵ*_2*ij*_, *ϵ*_3*ij*_, *ϵ*_4*ij*_, are independent normally distributed residual error terms with a mean of zero and a variance of *σ*^2^_1_, *σ*^2^_2_, *σ*^2^_3_, *σ*^2^_4_.

#### Covariate model

Available covariates were body weight, height, age, sex, smoking status, pregnancy (for Tanzanians patients), and concomitant medications. Reported concomitant medications were coded as moderate to strong inhibitors or inducers of the cytochrome P450 isoenzymes (CYP) mostly involved in the metabolism of the anti-malarials (Table 2) [31-40]. This information was based on report of self-medication prior inclusion and prescription during the study.

**Table 2:** Concomitant medications included in the models

**Drug**	**Metabolism**	**Concomitant medications taken by study patients**	**Effect†**	%*
Artemether	*CYP2B6* ‡ [33,34]			0%
	*CYP2C9*‡ [33,34]	Ibuprofen, pyrimethamine and quinine	Strong to moderate inhibitor	22%
	*CYP2C19*‡ [34]			0%
	*CYP3A4*[32-35]	Caffeine, doxycycline, erythromycine and metronidazole	Moderate inhibitors	10%
	*CYP3A5*[32,34]			0%
Dihydroartemisinin	Glucuronidation [36]	--		
Lumefantrine	*CYP3A4*[32,35]	Caffeine, doxycycline, erythromycine and metronidazole	Moderate inhibitors	10%
Mefloquine	*CYP3A4*[37-39]	Clarithromycine, caffeine, metronidazole and tetracycline	Strong to moderate inhibitor	8%
	*CYP3A5*[37]			0%
Piperaquine	Unknown [31]	Chloroquine, ibuprofen and quinine	Strong to moderate inhibitor of *CYP2C8*, *CYP2C9* and / or *CYP2D6*	45%

* Percentage of patients in the study taking the respective inhibitor.

† From UpToDate Online 17.1 (http://www.uptodateonline.com/online/index.do).

‡ Only *in vitro*, not seen in healthy volunteers [40].

The covariate analysis was performed using a stepwise insertion/deletion approach. Visual inspection of the correlation between *post hoc* individual estimates of the pharmacokinetic parameters and the available covariates was first conducted by graphical exploration. Potentially influential covariates were then incorporated sequentially into the pharmacokinetic model. The typical value of a given parameter *θ* (e.g., *CL*) was modeled to depend on the covariate (*X)* either linearly *θ* = *θ*_a_ × [1 + *θ*_*b*_ × *X*], exponentially *θ* = *θ*_*a*_^*X*^ or as an allometric power function $\theta =\theta_{a}\times X^{\theta_{b}}$, with *θ*_*a*_ representing the population value of the pharmacokinetic parameter and *θ*_*b*_ the contribution of the covariate *X*, centered on the mean value; categorical covariates were coded as 0 or 1. In the allometric power models, *θ*_*b*_ was either estimated or fixed to literature values, i.e. 0.75 for *CL* and 1 for *V*_*C*_[41]. At the end of the analysis, all patient characteristics showing an influence on the parameters were again confirmed by comparing the full model (with all factors included) to models from which each of the factors was removed sequentially.

#### Model selection and parameter estimation

NONMEM^®^[28] (version 6.0, NM-TRAN, version II) was used with the FOCE INTERACTION method to fit the data. The difference in the minimum objective function value (*Δ*OFV) provided by NONMEM^®^, (–2 log likelihood, approximate *χ*^2^ distribution) was used to discriminate between models using the likelihood ratio test. A model was considered superior to another nested model when the OF value was reduced by at least 3.84 points (*p* < 0.05). Covariate analysis comprised forward selection of influential factors followed by backward deletion. Covariates were retained in the final model at the statistical level of *p* < 0.01. Model assessment was based on diagnostic plots (goodness-of-fit plots) along with standard errors and correlation matrix of parameter estimates, size of residual errors and eta-shrinkage.

#### Model validation

The stability and the performance of the final population pharmacokinetic model were validated by the bootstrap method. Two hundred data sets were reconstructed by re-sampling from the original data using the Perl-speaks-NONMEM (PsN) Toolkit Version 3.2.4 [42,43]. The final population pharmacokinetic model was fitted repeatedly to the 200 bootstrapped samples and pharmacokinetic parameters were calculated for each dataset. The mean, standard error and 95% confidence interval of each parameter obtained from the bootstrap analysis were then compared to the corresponding parameters obtained with the original dataset. The statistical analysis was performed using PsN version 3.2.4 [44]. The final model was also validated using visual predictive check (VPC) obtained by simulation of data for 1’000 individuals based on the final model and generating 2.5^th^, 50^th^ and 97.5^th^ percentiles. The observed concentrations were plotted against the 95% prediction interval (P.I._95%_) of the simulated dataset at each time point and visually compared. Figures were generated using GraphPad Prism (Version 4.00 for Windows, GraphPad Software, San Diego California USA [45]).

#### Model-based simulations for lumefantrine

Concentration-time profiles of lumefantrine in 1’000 individuals receiving two different 6-dose regimens over 3 days (doses at 0, 8, 20, 32, 44 and 56 h) and 5 days (doses at 0, 8, 24, 48, 72, 96 h) were performed based on the final model including inter-patient variability. These simulations served to purpose of quantifying the percentage of patients at day 7 below the different cut-off thresholds of 50 ng/ml, 175 ng/ml, 280 ng/ml and 600 ng/ml associated with treatment outcome [6]. In addition, the simulation-based predicted median time (95% P.I.), estimated from time of last dose to 168 h (day 7), at which patients would exhibit concentrations below the cut-off values of 50 ng/ml, 175 ng/ml and 280 ng/ml was derived.

## Results

### Population pharmacokinetic analyses

Patients’ baseline characteristics are summarized in Table 3. The median (range) of samples available per subject was 3 (2–4) for LF, 3 (1–4) for DLF, 2 (1–3) for AM and 2 (1–3) for DHA in Tanzania, 5 (3–6) for MQ, 1 for AS and 1 for DHA in Phnom Dék and 5 (4–6) for PPQ and 1 for DHA in Pramoy. The number of measured samples per time point is presented in Additional file 1.

**Table 3:** Patients’ characteristics at inclusion

	Kibaoni (Tanzania)*	
**Characteristic**	**AM / DHA†**	**LF / DLF†**
Total patients	135	143
Sex male / female (%)	56 (41) / 79 (59)	62 (43) / 81 (57)
Age median (range) [years]	10 (1–78)	9 (1–78)
Body weight median (range) [kg]	20 (6.5–150)	20 (6.5–150)
Height median (range) [cm]	126 (52–181)	126 (52–181)
Pregnancy (%)	3 (2)	3 (2)
Smokers (%)	1 (1)	1 (1)
Median time sick (range) [days]	3 (1–14)	3 (1–14)
Median body temperature (range) [°C]	37.5 (35.2–40.4)	37.6 (35.2–40.4)
Median asexual parasites (range) [μL^-1^]	15,360 (120– > 399,960)^a^	15,360 (120– > 399,960)^a^
Median respiratory rate (range) [min^-1^]	24 (16–38)	24 (16–38)
Median hematrocrit (range) [%]	N.A.	N.A.
Median haemoglobin (range) [g/dL]	10.5 (5.1–16.3)	10.4 (5.1–16.3)
	**Phnom Dék (Cambodia)**	
**Characteristic**	**AS / DHA† and MQ**	
Total patients	63	
Sex male / female (%)	37 (59) / 26 (41)	
Age median (range) [years]	18 (2–57)	
Body weight median (range) [kg]	43 (10.5–66)	
Height median (range) [cm]	153 (73–172)	
Pregnancy (%)	N.A.	
Smokers (%)	17 (27)	
Median time sick (range) [days]	2 (2–3)	
Median body temperature (range) [°C]	38.6 (37.9–40.4)	
Median asexual parasites (range) [μL^-1^]	19,600 (1,200–160,000)	
Median respiratory rate (range) [min^-1^]	28 (20–38)	
Median hematrocrit (range) [%]	30 (24–37)	
Median haemoglobin (range) [g/dL]	N.A.	
	Pramoy (Cambodia)*	
**Characteristic**	**DHA**	**PPQ**
Total patients	56	60
Sex male / female (%)	34 (61) / 22 (39)	38 (63) / 22 (37)
Age median (range) [years]	18 (7–53)	18 (7–53)
Body weight median (range) [kg]	42 (15–67)	42 (15–67)
Height median (range) [cm]	151 (105–171)	152 (105–171)
Pregnancy (%)	N.A.	N.A.
Smokers (%)	12 (21)	14 (23)
Median time sick (range) [days]	2 (1–3)	2 (1–3)
Median body temperature (range) [°C]	38.4 (37.8–39.8)	38.4 (37.8–39.8)
Median asexual parasites (range) [μL^-1^]	16,858 (1038–219,333)	17,229 (1038–219,333)
Median respiratory rate (range) [min^-1^]	28 (20–40)	28 (20–40)
Median hematrocrit (range) [%]	41 (30–50)	41 (30–50)
Median haemoglobin (range) [g/dL]	N.A.	N.A.

*Abbreviations: AM* artemether, *AS* artesunate, *DHA* dihydroartemisinin, *LF* lumefantrine, *MQ* mefloquine, *PPQ* piperaquine.

* The number of patients included for the final analysis was not equal for the artemisinin-derivative and the partner drug.

† Parent drug / metabolite.

^a^ Asexual parasites were counted against 200 white blood cells and converted to parasites/μL by assuming a density of 8,000 white blood cells/μL.

### Artemether

A one-compartment model with first-order absorption from the gastrointestinal tract adequately described the data; no improvement was obtained with a two-compartment model (difference in the objective function (*Δ*OFV) = 0). For this drug, no baseline residual plasma concentrations were measured. In addition to *CL*, the assignment of an inter-patient variability on *k*_a_ (but on no other parameter) significantly improved the description of the data (*Δ*OFV = −63). Univariate analyses showed that body weight (*Δ*OFV ≤ −45.1), age (*Δ*OFV ≤ −49.0), height (*Δ*OFV ≤ −42.7) and sex (*Δ*OFV ≤ −13.3) significantly influenced *CL*. In multivariate analyses, only body weight remained significant since all other variables were correlated to body weight. Linear and allometric power functions described the effect of body weight on *CL* similarly well (no statistical difference was observed between the two models (*Δ*OFV = −2)); the latter was finally chosen based on goodness of fit plots. The exponent of the allometric power function was estimated to be 0.66 and finally fixed to the literature value (0.75), since statistically not different (*Δ*OFV = 1). Inhibitors of *CYP2C9* and/or *CYP3A4* significantly influenced *CL* as well (*Δ*OFV = −7), indicating a 70% decrease in *CL* in patients exposed to either a *CYP2C9* or *CYP3A4* inhibitor. Multivariate analysis showed an additive influence of body weight and CYP inhibitors on CL (*Δ*OFV = −61 relative to the model without covariates).

**Figure 3:**
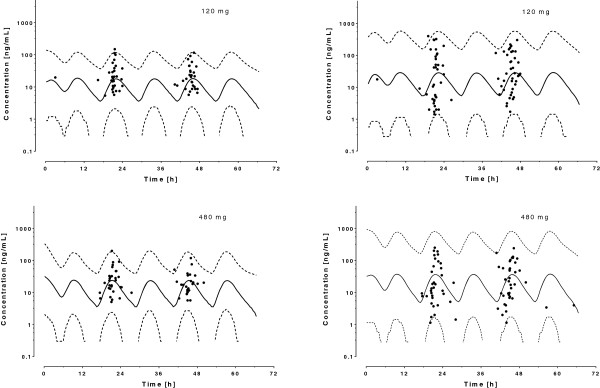
**Observed plasma concentrations of artemether (left panels) and dihydroartemisinin (right panels) after administration of 6 ×** **20 = 120 mg (children) and 6 ×** **80 = 480 mg (adults) artemether in 135 Tanzanian patients.** The solid lines represent the mean population prediction and the dotted lines 95% prediction intervals. Triangles and squares represent residual plasma concentrations of lumefantrine and desbutyl-lumefantrine found prior treatment initiation.

Metabolite concentrations (DHA) were included in the model using an additional compartment, assuming linear metabolism and elimination. The assignment of an inter-patient variability on the metabolism rate constant *k*_*23*_ yielded a better fit of the data (*Δ*OFV = −81), while no improvement was observed when assigning variability to the metabolite clearance *CL*_*met*_ (*Δ*OFV = 0). Finally, none of the available covariates significantly affected DHA pharmacokinetics. A proportional error model for drug and metabolite provided the best description of intra-patient variability. The parameter estimates for the final model and derived parameters are in Table 4. The concentration-time plots of AM and DHA in the 135 patients included in the analysis with average population predictions and 95% prediction intervals is presented in Figure 3.

**Table 4:** Final population parameter estimates of artemether, lumefantrine, mefloquine and piperaquine and estimates from the bootstrap evaluation in 200 replicates

**Population analysis**					**Bootstrap evaluation**		
**Parameter**	**Estimate**	**S.E.**^**a**^	**IIV**^**b**^	**S.E.**^**c**^	**Mean of 200**	**S.E.**	**95% C.I.**^**d**^
**Artemether**							
*CL* [L/h/kg]	24.7 × BW^0.75^	10%	44%	17%	24.4 × BW^0.75^	10%	19–29
*θ*_*INH*_	−0.3	45%			−0.27	60%	0.5–1.1
*V*_*C*_ [L/kg]	129	20%			133	26%	88–232
*V*_*M*_ [L]	Fixed to *V*_*C*_						
*k*_a_ [h^-1^]	0.27	11%	119%	11%	0.27	14%	0.21–0.35
*k*_23_ [h^-1^]	5.86	21%	68%	9%	5.83	25%	3.6–9.7
*CL*_*met*_ [L/h]	419	30%			440	42%	213–927
*σ*_*C*_ (CV%)	74%	9% ^c^			73%	11% ^c^	59%–86%
*σ*_*M*_ (CV%)	119%	11% ^c^			116%	9% ^c^	88%–149%
**Lumefantrine**							
*CL* [L/h/kg]	$0.84\times {\mathrm{BW}}^{\theta_{\mathrm{BWCL}}}$	28%	38%	14%	$0.87\times {\mathrm{BW}}^{\theta_{\mathrm{BWCL}}}$	24%	0.51-1.37
*θ*_*BWCL*_	0.52	19%			0.51	14%	0.36-0.65
*V*_*C*_ [L/kg]	$59.9\times {\mathrm{BW}}^{\theta_{\mathrm{BWCL}}}$	28%	33%	11%	$59.5\times {\mathrm{BW}}^{\theta_{\mathrm{BWCL}}}$	24%	35.1–91.9
*θ*_*BWVC*_	0.35	28%			0.34	19%	0.19-0.45
*V*_*M*_ [L]	Fixed to *V*_*C*_						
*k*_*a*_ [h^-1^]	0.54	31%			0.48	43%	0.11–0.88
*F*_0_ [mg]	2.53	14%	103%	14%	2.45	15%	1.58–3.28
*k*_23_ [h^-1^]	3.7 × 10^-4^	12%	38%	15%	3.7 × 10^-4^	9%	(3.0–4.4) × 10^-4^
*CL*_*met*_ [L/h]	4.8	10%			4.6	13%	3.4–5.7
*σ*_*C*_ (CV%)	60%	9% ^c^			61%	31%	55%–77%
*σ*_*M*_ [μmolL^-1^]	0.013	4% ^c^			0.013	45%	0.010–0.016
**Mefloquine**							
*CL* [L/h/kg]	0.10 × BW^0.75^	5%	12%	88%	0.10 × BW^0.75^	5%	(0.09–0.11)
*V*_*C*_ [L/kg]	8.93 × BW	6%	19%	96%	9.01 × BW	6%	(8.04–10.20)
*k*_a_ [h^-1^]	0.15	14%			0.15	14%	0.12–0.19
*F*_0_ [mg]	33.1	56%	175%	48%	31.0	43%	11.8–48.1
*σ*_*C*_ [μmolL^-1^]	43%	6% ^c^			43%	6% ^c^	0.14–0.22
**Piperaquine**							
*CL* [L/h/kg]	4.50 × BW^0.75^	13%	45%	61%	4.26 × BW^0.75^	22%	(3.24–5.76)
*V*_*C*_ [L/kg]	346 × BW	12%	65%	48%	347 × BW	13%	(260–432)
*Q* [L/h]	122	13%			126	13%	86–158
*V*_*P*_ [L]	18,600	22%	50%	77%	20,053	37%	8,778–28,422
*k*_a_ [h^-1^]	0.93	28%			1.00	34%	0.35–1.52
*F*_0_ [mg]	123	18%			125	18%	75–171
*σ*_*C*_ [μmolL^-1^]	41%	10% ^c^			41%	6% ^c^	0.14–0.21

*Abbreviations: CL* clearance, *BW* body weight, *θ*_*INH*_ inhibitors effect (CYP3A4 and/or CYP2C19) on *CL* expressed as *(1- θ*_*INH*_ × *INH)*, *V*_*C*_ central volume of distribution, *Q* inter-compartment clearance, *V*_*M*_ volume of distribution of the metabolite, *V*_*P*_ peripheral volume of distribution, *k*_*a*_ first-order absorption rate constant, *F*_0_ residual amount from the previous treatment, *k*_23_ metabolism rate constant, *CL*_met_ metabolite clearance, *σ*_*C*_ exponential residual error for the central compartment, *σ*_*M*_: exponential residual error for the metabolite compartment.

^a^ Standard error (S.E.) of the estimate *θ*_*i*_ defined as S.E estimate/estimate, expressed as a percentage.

^b^ Inter-individual variability.

^c^ Standard error (S.E.) of the coefficient of variation defined as √S.E estimate/estimate, expressed as a percentage.

^d^ 95% confidence interval (C.I.).

### Lumefantrine

**Figure 4:**
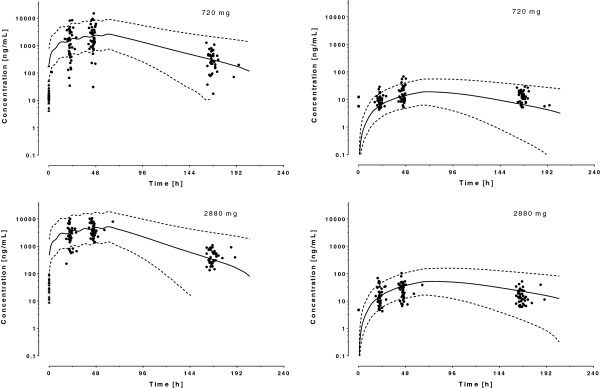
**Observed plasma concentrations of lumefantrine (left panels) and desbutyl-lumefantrine (right panels) after administration of 6** × **120 = 720 mg (children) and 6** × **480 = 2880 mg (adults) lumefantrine in 135 Tanzanian patients.** The solid lines represent the mean population prediction and the dotted lines 95% prediction intervals. Triangles and squares represent residual plasma concentrations of lumefantrine and desbutyl-lumefantrine found prior treatment initiation.

A one-compartment model with first-order absorption from the gastrointestinal tract and linear metabolism into DLF described adequately the data; a two-compartment model for LF or for DLF did not improve the model fit (*Δ*OFV = 0). The average estimated residual dose from previous treatments (*F*_*0*_) was 1.6 mg, which corresponds to 0.3–1.3% of the recommended LF first dose (120–480 mg). Adding an inter-patient variability on *V*_*C*_ (*Δ*OFV = −75), *k*_23_ (*Δ*OFV = −199) and *F*_0_ (*Δ*OFV = −17) in addition to *CL* improved the description of the data, but no variability on the other parameters was significant. A proportional error model best described the residual intra-patient variability for LF and an additive one for DLF. Inclusion of age, height and body weight on both *CL* and *V*_*C*_ improved the fit (*Δ*OFV ≤ −30). Since age, height and body weight were correlated, only body weight was retained for further testing. Linear and allometric power functions adequately described its influence on *CL* and *V*_*C*_ equally well (*Δ*OFV ≤ −34); the latter was selected based on visual inspection of graphical analysis. The estimations of the exponents of the allometric power functions were 0.52 and 0.35 for *CL* and *V*_*C*_, respectively, and provided a better fit than the fixed literature values (*Δ*OFV ≤ −17). Sex, smoking status, pregnancy and concomitant medications did not affect *CL* or *V*_*C*_ (*Δ*OFV ≥ −0.2). The parameter estimates for the final model and derived parameters are given in Table 4. Figure 4 shows the concentration-time plots of LF and DLF in the 143 patients included in the analysis with average population predictions and 95% prediction intervals.

### Mefloquine

**Figure 5:**
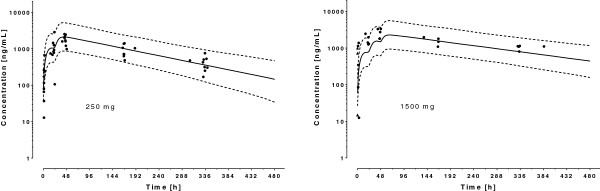
**Observed mefloquine plasma concentration after administration of a 2 ×** **125 = 250 mg (children) and 3 ×** **500 = 1500 mg (adults) dose in 63 Cambodian patients.** The solid lines represent the mean population prediction the dotted lines the 95% prediction intervals. Triangles represent residual plasma concentrations of mefloquine or piperaquine found prior treatment initiation.

A one-compartment model with first-order absorption from the gastrointestinal tract appropriately described the data, with no improvement using a two-compartment model (*Δ*OFV = 0). For this drug, the residual dose from previous treatments (*F*_0_) was estimated to be 33.1 mg, corresponding to 6.7–26.7% of an initial dose of 125–500 mg. A better fit was obtained by assigning an inter-patient variability on *V*_*C*_ (*Δ*OFV = −172) and *F*_0_ (*Δ*OFV = −211) in addition to *CL*. The use of a proportional error model for the residual intra-patient variability fitted the data well. Again, inclusion of age, height, body weight and sex improved the fit in univariate analyses (*Δ*OFV ≤ −136). Plots of *CL* and *V*_*C*_ as a function of body weight suggested that an allometric power function, with exponent fixed to literature values, should be preferred to a linear relationship. The addition of smoking status and concomitant medications on *CL* and *V*_*C*_ did not improve the model significantly (*Δ*OFV ≥ −1.2). Multivariate analysis indicated that body weight remained the unique significant covariate on both *CL* and *V*_*C*_. The parameter estimates for the final model and derived parameters are given in Table 4. Figure 5 depicts the simulated plasma concentration-time plot of MQ in the 63 patients included in the analysis with average population predictions and 95% prediction intervals.

### Piperaquine

**Figure 6:**
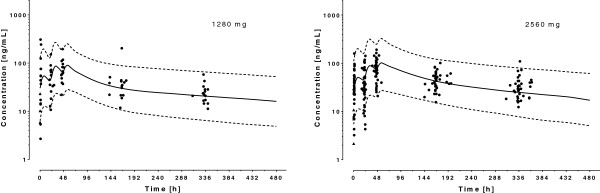
**Observed piperaquine plasma concentrations after administration of 2 × 480 + 320 = 1280 mg (children) and 2 × 960 + 640 = 2560 mg (adults) dose in 60 Cambodian patients.** The solid lines represent the mean population prediction the dotted lines the 95% prediction intervals. Triangles represent residual plasma concentrations of mefloquine or piperaquine found prior treatment initiation.

A two-compartment model with first-order absorption from the gastrointestinal tract described the data better than a one-compartment model (*Δ*OFV = −97), but no additional benefit was seen with a three-compartment model (*Δ*OFV = 0). The residual dose of PPQ was estimated to be 123 mg, which corresponds to 12.8–25.6% of an initial dose of 480–960 mg. Assigning an inter-patient variability on *V*_*C*_ (*Δ*OFV = −129) and *V*_*P*_ (*Δ*OFV = −17) in addition to *CL* improved the fit and the use of a proportional error model for the residual intra-patient variability fitted the data adequately. *CL* and *V*_*C*_ were again influenced by body weight (*Δ*OFV = −14 and −27, respectively); the relationship was best described using an allometric power function with exponents fixed to the literature values, and was not statistically different from estimated values (*Δ*OFV ≥ −2). Addition of sex or smoking status as covariates of *CL* did not improve the model fit (*Δ*OFV ≥ −2). As the metabolizing CYP of PPQ are not known and few concomitant treatments were reported, this variable was not included in the model. The parameter estimates for the final model and derived parameters are given in Table 4. Figure 6 shows the simulated plasma concentration-time plot of PPQ in the 60 patients included in the analysis with average population predictions and 95% prediction intervals.

### Concentration-time simulations of lumefantrine

**Figure 7:**
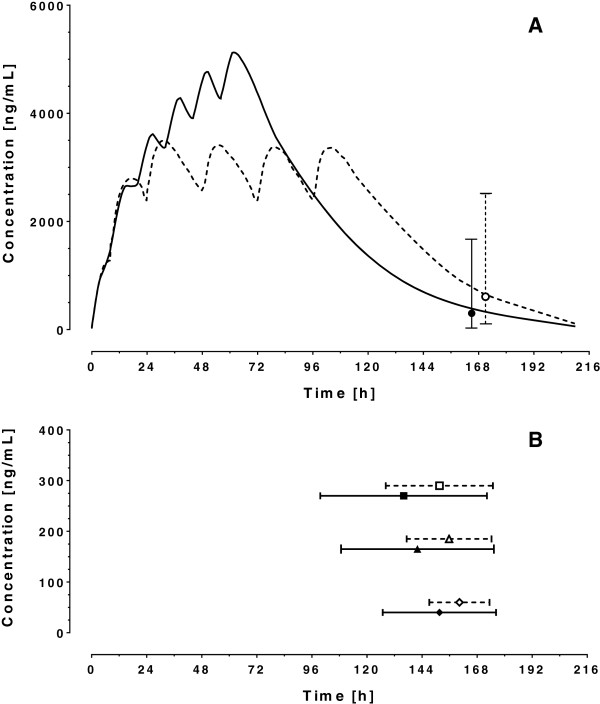
**Concentration-time simulations of lumefantrine. A:** Predicted median concentration of lumefantrine after administration of 6 × 480 mg (adults) regimen over 3 (continuous line) and 5 days (dotted line). Day 7 (168 h) median predicted concentrations (circles) with their 95% prediction intervals are shown for the two dosage regimens. **B**: Predicted mean (95% C.I.) time (estimated from time of last dose to 168 h) at which concentrations lie below the cut-off values of 50 ng/ml (rhombi), 175 ng/ml (triangles) and 280 ng/ml (squares). Full and empty symbols associated with continuous and dotted lines represent 6-dose regimens over 3 and 5 days respectively.

The day 7 predicted median concentrations of lumefantrine after administration of a 6 dose-regimen over 3 days were 300.9 ng/ml (P.I._95%_ 12.2−2015.0 ng/ml). Considering the large inter-patient variability in LF kinetics, 11% of the patients would exhibit day 7 concentrations below the cut-off of 50 ng/ml, 33% below 175 ng/ml, 48% below 280 ng/ml and 71% below 600 ng/ml. Prolonging the time of drug administration over 5 days would provide median concentrations of 608.7 ng/ml (P.I._95%_ 69.5−3515 ng/ml), with 1%, 10%, 21% and 49% of patients with concentrations below the cut-off of 50 ng/ml, 175 ng/ml, 280 ng/ml and 600 ng/ml, respectively. In addition, simulations predicted that patients would exhibit concentrations below the cut-off values of 50 ng/ml, 175 ng/ml and 280 ng/ml in a median (95% P.I.) of 152 h (126.8−176.3 h), 142 h (108.7−175.3 h) 136 h (99.7−172.3 h), respectively after a standard dosing regimen of 6 doses over 3 days. Increasing the 6-dose regimen over 5 days would increase the median time to 160 h (147.2−173.4 h), to 156 h (137.3−174.3 h) and to 152 h (128.2−174.9 h) for the 3 proposed cut-off values, respectively (Figure 7).

## Discussion

This study describes the disposition of three widely used forms of ACT (namely; AM-LF, AS-MQ and DHA-PPQ) and explores factors potentially influencing the marked variability in drug exposure. The estimated values of clearance and volume of distribution for AM (including its metabolite DHA), LF (including its desbutyl metabolite DLF), MQ and PPQ are in line with previously published results (see Table 5 for review), so are the large inter-patient and marked intra-individual variability [8,32,46-55]. Below some of the key findings are discussed.

**Table 5:** **Population estimate of clearance ( *CL *) and steady-state volume of distribution (*V***_***SS***_**) of antimalarials for a person with a median body weight of 70 kg from mixed effects models from the present study and in previous studies**

**Drug**	**Subjects**	**Median age (range) [years]**	**No. of patients**	**Duration of sampling**	***CL *[L/h]**	***V***_***SS ***_**[L]**	**Ref.**
AM	Patients	10 (1–78)	135	7 days	598	129	Present study
	Patients	22 (14–60)	217	360 h	252^a^	304^a^	[47]
	Patients	4.0 (1–10)	50	72 h	182–700^b^	364	[46]
	Patients	7.7 ± 1.4^c^	13	28 days	102	1263	[48]
	Pregnant women	21 (16–35)	21	10 h	1054^d^	2602^d^	[49]
DHA^e^	Patients	10 (1–78)	135	7 days	419	129^f^	Present study
	Patients	22 (14–60)	217	360 h	237^a^	147^a^	[47]
	Pregnant women	21 (16–35)	21	10 h	564	69	[49]
LF	Patients	9 (1–78)	143	7 days	7.7	265^f^	Present study
	Patients	22 (14–60)	217	–	21	301	[47]
	Patients	23 (13–59)	102	–	7	298	[32]
	Patients	20 (5–66)	309	–	7.6	361	[32]
	Pregnant women	24 (15–42)	103	336 h	8.7	257	[50]
	Patients	4.0 (1–10)	50	72 h	5.4	623	[46]
	Patients	7.7 ± 1.4^c^	13	28 days	7.29	506	[48]
DLF^g^	Patients	9 (1–78)	143	7 days	336	265^f^	Present study
	Patients	7.7 ± 1.4^c^	13	28 days	701	119’500	[48]
MQ	Patients	18 (2–57)	63	14 days	2.4	625	Present study
	Patients	14.8 (8–61)	128	28 days	1.4	574.7	[8]
	Patients	9.3 (4–15)	74	28 days	3.71	1,089.2	[8]
	Prophylaxis	26^h^ (18–55)	1,111	26 weeks	1.75	863	[51]
	Patients	19 (2–55)	50	63 days	2.1	767.62	[52]
PPQ	Patients	18 (7–53)	60	14 days	109	42,820	Present study
	Patients	3–55	98	63 days	98^h^	61,180^i^	[53]
	Patients	6 (2–10)	236	42 days	29	14,972	[54]
	Pregnant women	25 (18–43)	24	84 days	90	37,030	[55]
	Non-pregnant women	27.5 (18–45)	24	84 days	92	58,030	[55]

*Abbreviations: AM* artemether, AS artesunate, DBL desbutyl-lumefantrine, DHA dihydroartemisinin, LF lumefantrine, MQ mefloquine, PPQ piperaquine.

^a^ Fixed parameter at mean value.

^b^ From first to sixth dose.

^c^ Mean ± standard deviation.

^d^ Parameter estimates are weight normalized based on published population mean values divided by the mean weight of subjects.

^e^ Only as metabolite after administration of AM.

^f^ Fixed to *Vss* of AM.

^g^ Only as metabolite after administration of LF.

^h^ Mean.

^i^ Population estimate for a patient with a median body weight of 48 kg.

### Prior treatments

Interestingly and worryingly, more than half of the patients had residual concentrations above the lower limit of quantification of at least one antimalarial on admission (74.3% in Tanzania Kibaoni, 51.6% in Cambodia Phnom Dék and 68.9% in Cambodia Pramoy). Residual doses were low in Tanzania for LF, but much higher values were estimated in Cambodia for MQ and PPQ, with up to approximately one quarter of an initial dose already present at baseline. The levels reflect the different residence times of these drugs; the proportions show the extent of unregulated drug use and selective pressure going on in these countries [29,30].

### Determinants of exposure – implications for dosing

There was a clear correlation between *CL*, *V*_*C*_ and body weight for all drugs, which accounted for about 10−30% of the inter-individual variability in these two parameters. Body weight was highly correlated with age, sex and height and remained the only significant parameter in the multivariate analyses. This result supports the use of antimalarial dosing regimens based on body weight, or age as a proxy for it. What the model cannot predict is whether an additional correction to dosing should be made for children (or naïve adults) on account of lack of immunity.

The scaling factor of 0.75 for *CL* and 1 for *V*_*C*_ described the relationships with body weight adequately, with the exception of LF for which the usual allometric scaling function provided a worse description of the data. Although a controversy persists regarding the body weight-dependent allometric exponent in the literature, it is not clear whether a different scaling between children and adults should be expected for this specific drug, or whether some confounding factors (different compliance between adult and children, different food intake) could have contributed to this finding.

### Interactions and metabolism

Most of the anti-malarial drugs are metabolized by CYPs and concomitant treatment with inhibitors or inducers of these enzymes might, therefore, influence their elimination. This study detected only an influence of *CYP2C9* and *CYP3A4* inhibitors on AM clearance, which was decreased by 70% in patients with concomitant treatment. The fact that very few co-medications were reported might explain the lack of interactions for LF, MQ or PPQ. For the latter, the metabolizing pathways are not known*.* Among other factors, genetic polymorphisms in the enzymes responsible for antimalarial drugs could represent another important source of variability. A population genetic- and pharmacokinetic-based analysis was conducted to address this issue and published elsewhere [27].

### Other conditions potentially influencing exposure

Pregnancy is known to lower blood concentrations of AM and LF, thus putting pregnant women at risk of under-dosing [31]. This study enrolled only three pregnant women in Tanzania, which prevented estimating the influence of pregnancy on LF or AM drug levels.

Food intake has been shown to affect strongly the bioavailability of LF, MQ and PPQ [56-59]. In practice, this is a source of systematic under-dosing; in a study of the adherence to treatment regimens in Tanzania, only 0.4% of patients were reported to take their antimalarials with food [60]. While in the present study patients admitted to the health facility were provided with food, outpatients were advised to eat directly before or after supervised drug-intake but adherence to this recommendation was based on self-reporting only, which, against the background of the above-mentioned adherence study, made it unreliable to have food effect included in the analysis. The absence of food information represents, however, a clear limitation of this study.

### Structural and variance model

These drugs are known to exhibit multi-compartmental pharmacokinetics (two- or three-compartment disposition model) that could not be well captured owing to the limited duration of sampling compared to other studies. Although our data could provide a good estimation of *CL* and variability, appropriate description of terminal elimination phase could not be done. In addition, due to the very sparse sampling design during the absorption phase, no estimation of different absorption model could be performed, neither could the variability in the absorption quantified for LF, MQ and PPQ, with the exception of AM that exhibited a large inter-patient variability in its absorption. This large variability could result from both inherent characteristics of the drug [46,61], and practical issues with dosing using crushed AM-LF pills (the paediatric formulation was not available for this study) [62]. Another limitation of this study is that no estimation could be made for AS and DHA for the AS-MQ and DHA-PPQ treatments.

### Simulations for LF

While the notion of concentration-effect relationship for LF is generally accepted, there is yet no common understanding of what the therapeutic target concentration should be. The published day 7 LF concentrations associated with therapeutic response range from 175 ng/ml to 600 ng/ml [6]. A recent, large pooled analysis of LF concentration-efficacy data confirmed that a strong association exists between low day 7 LF concentrations and an increased risk of recurrence until day 42, and until day 21 for new infection [7]. However, the authors of the pooled analysis concluded that there is no clear cut-off value for the thresholds associated with risk of recrudescence or new infection, but that cut-offs can be defined based on achieving a proportion of the desirable effect. For example, in low transmission areas a cut-off of 125 ng/ml gave efficacy rates of 84% and 96% at 42 days, and in high transmission areas a cut-off of 50 ng/ml gave efficacy rates of 80% and 95% at 42 days. In the Tanzanian sample of this study, 35% of the patients had a concentration below the cut-off value of 175 ng/ml, but only one of the 7 patients who had recurrent parasitaemia (unadjusted failure rate) was in this group.

Owing to the important variability in LF pharmacokinetics, the simulations under the standard 6-dose over 3 days schedule shows that a substantial proportion of the patients would present concentrations below the various proposed therapeutic targets at day 7. The considerable inter-individual variability in LF plasma concentrations additionally suggests that in some patients plasma LF concentrations would fall below the proposed minimal concentrations between the fourth and seventh day (3^rd^ and 4^th^ cycles) after treatment (i.e. before all the parasite had been eradicated). Splitting the same recommended total dose over 5 days would greatly reduce the probability of exhibiting sub-therapeutic drug concentrations, as already shown by other studies [32,63]. However, in practice, the potential increased exposure with this 5-day regimen may be impeded by the possible risk of lower adherence to the treatment. Very little evidence exists for the other compounds. For MQ, the time over the MIC seems an important component associated with treatment efficacy [8]. Our results indicate that this drug exhibits the least variability in its disposition and it is thus not expected that differences in response would be strongly related to variable drug levels.

## Abbreviations

ΔOFV: Difference in the minimum objective function value; θ: Typical value of given parameter in the population; ACT: Artemisinin-based combination therapy; AM: Artemether; AQ: Amodiaquine; AS: Artesunate; C.I.95%: 95% confidence interval; CL: Systemic clearance; CLmet: Metabolite clearance; CYP: Cytochrom P450 isoenzyme; DHA: Dihydroartemisinin; DLF: Debutyl-lumefantrine; F0: Residual amount of drug from the previous treatment; IIV: Inter-individual variability; k23: Metabolism rate constant from drug compartment to metabolite compartment; ka: Absorption rate constant; LF: Lumefantrine; MIC: Minimal inhibitory concentration; MQ: Mefloquine; P.: *Plasmodium*; P.I.95%: 95% prediction interval; PN: Pyronaridine; PPQ: Piperaquine; PsN: Perl-speaks-NONMEM; Q: Inter-compartmental clearance; S.E.: Standard error; SP: Sulfadoxine-pyrimethamine; VC: Central volume of distribution; VM: Volume of distribution of the metabolites; VP: Peripheral volume of distribution; VPC: Visual predictive check; VSS: Steady-state volume of distribution; WHO: World Health Organization; WWARN: World-Wide Antimalarial Resistance Network; X: Covariate.

## Competing interests

Support for this work was provided by grant No 320000-112479 from the Swiss National Science Foundation. The authors declare that they have no competing interests.

## Authors’ contributions

EMSH contributed to the design of the study, the acquisition of patient samples and laboratory data, the analysis and interpretation of data and to drafting of the manuscript. DS and AMK made substantial contributions to the acquisition of patient samples and the coordination of the study. FA was involved in the acquisition of patient samples and the assay development for the pharmacogenetic analysis. BZ, TM and LAD where involved in the assay development and in the quantitation of the drug concentration levels. MG and CC carried out the population pharmacokinetic analysis and made substantial contributions to interpretation of data and to drafting and revising the manuscript. TB contributed to interpretation of pharmacokinetic data and the revising of the manuscript. BG, HPB and PO conceived the study, participated in its design and coordination, helped to draft the manuscript and revised it critically for important intellectual content. All authors have given final approval of the version to be published.

## Authors’ information

The opinions expressed in this paper are those of the authors and may not reflect those of their employing organizations. PO is a staff member of the WHO; the authors alone are responsible for the views expressed in this publication and they do not necessarily represent the decisions, policy or views of the WHO.

## Supplementary Material

Additional file 1 **Number of samples per time point.** The table provided summarizes the number of patient samples included in the population pharmacokinetic model of each anti-malarial drug (and its metabolite where applicable) for every sampling time point. Click here for file
