# Supplementary material for: Population pharmacokinetics of mefloquine, piperaquine and artemether-lumefantrine in Cambodian and Tanzanian malaria patients
Source: Malar J. 2013 Jul 10;12:235. doi: 10.1186/1475-2875-12-235 (PMC3720542; doi:10.1186/1475-2875-12-235)
Supplement: Additional file 1 — Number of samples per time point. The table provided summarizes the number of patient samples included in the population pharmacokinetic model of each anti-malarial drug (and its metabolite where applicable) for every sampling time point. [file 1475-2875-12-235-S1.pdf]

Table S1 Number of samples per time point

Day	AM / DHA†	LF / DLF†	AS / DHA†	MQ	DHA	PPQ
Pre-treatment		78 / 7		16		9
0	1 / 1	1 / 0	58 / 62	62	56	57
1	103 / 120	138 / 123	1 / 1	64		59
2	91 / 129	137 / 133		62		60
3	0 / 1	1 / 1				
6				4		6
7		125 / 123		50		48
8		5 / 5		4		5
9		1 / 1		1		
13				6		3
14				46		49
15				4		6
16				2		

Drugs used: AM: artemether; AS: artesunate; DHA: dihydroartemisinin; LF: lumefantrine; MQ: mefloquine; PPQ: piperaquine.

† Parent drug / metabolite
